# Engineering Useful Microbial Species for Pharmaceutical Applications

**DOI:** 10.3390/microorganisms13030599

**Published:** 2025-03-05

**Authors:** Amankeldi K. Sadanov, Baiken B. Baimakhanova, Saltanat E. Orasymbet, Irina A. Ratnikova, Zere Z. Turlybaeva, Gul B. Baimakhanova, Aigul A. Amitova, Anel A. Omirbekova, Gulzat S. Aitkaliyeva, Bekzhan D. Kossalbayev, Ayaz M. Belkozhayev

**Affiliations:** 1 LLP “Research and Production Center for Microbiology and Virology”, Almaty 050010, Kazakhstan; a.sadanov1951@gmail.com (A.K.S.); bbbayken@mail.ru (B.B.B.); s_orazymbet@inbox.ru (S.E.O.); iratnikova@list.ru (I.A.R.); tzj2009@yandex.kz (Z.Z.T.);; 2 Department of Chemical and Biochemical Engineering, Geology and Oil-Gas Business Institute Named After K. Turyssov, Satbayev University, Almaty 050043, Kazakhstan; g.aitkaliyeva@satbayev.university (G.S.A.); belkozhayev1991@gmail.com (A.M.B.); 3 Faculty of Biology and Biotechnology, Al-Farabi Kazakh National University, Almaty 050040, Kazakhstan; anel.omirbekova@kaznu.edu.kz; 4 Ecology Research Institute, Khoja Akhmet Yassawi International Kazakh-Turkish University, Turkistan 161200, Kazakhstan

**Keywords:** microbial engineering, synthetic biology, biopharmaceuticals, machine learning, artificial intelligence

## Abstract

Microbial engineering has made a significant breakthrough in pharmaceutical biotechnology, greatly expanding the production of biologically active compounds, therapeutic proteins, and novel drug candidates. Recent advancements in genetic engineering, synthetic biology, and adaptive evolution have contributed to the optimization of microbial strains for pharmaceutical applications, playing a crucial role in enhancing their productivity and stability. The CRISPR-Cas system is widely utilized as a precise genome modification tool, enabling the enhancement of metabolite biosynthesis and the activation of synthetic biological pathways. Additionally, synthetic biology approaches allow for the targeted design of microorganisms with improved metabolic efficiency and therapeutic potential, thereby accelerating the development of new pharmaceutical products. The integration of artificial intelligence (AI) and machine learning (ML) plays a vital role in further advancing microbial engineering by predicting metabolic network interactions, optimizing bioprocesses, and accelerating the drug discovery process. However, challenges such as the efficient optimization of metabolic pathways, ensuring sustainable industrial-scale production, and meeting international regulatory requirements remain critical barriers in the field. Furthermore, to mitigate potential risks, it is essential to develop stringent biocontainment strategies and implement appropriate regulatory oversight. This review comprehensively examines recent innovations in microbial engineering, analyzing key technological advancements, regulatory challenges, and future development perspectives.

## 1. Introduction

Microorganisms have been recognized as invaluable resources for the pharmaceutical industry, playing a pivotal role in providing a wide range of bioactive compounds and biotechnological tools [1,2]. The advancement of genetic engineering and synthetic biology has facilitated the development and utilization of microbial species for the production of complex pharmaceutical compounds, ranging from antibiotics to biologics [3,4]. For example, species of *Streptomyces* have been genetically modified to produce various antibiotics with improved yields [5,6]. Additionally, model organisms such as *Escherichia coli* and *Saccharomyces cerevisiae* are widely utilized as primary bioreactors for the production of therapeutic proteins, including insulin, monoclonal antibodies, and other biologics [7,8]. These advancements are achieved through the optimization of genetic pathways, the enhancement of enzymatic activity, and the refinement of expression systems, facilitating the commercial-scale production of pharmaceutical products. Established approaches, such as adaptive evolution, continue to enhance the functional capabilities of microbial systems by promoting the selection of desirable phenotypic traits under carefully controlled environmental conditions. Furthermore, AI and ML technologies are widely utilized to effectively optimize engineering processes, accurately predict the dynamics of metabolic processes, and innovatively design biosynthetic pathways with unprecedented precision [9,10]. These approaches facilitate the transformation of engineered microbial systems into sustainable and efficient biotechnological production platforms. However, despite recent advancements, challenges such as optimizing metabolic pathways, scaling up production processes, and ensuring regulatory compliance remain significant barriers to translating microbial engineering innovations into clinical applications [11,12,13]. Although microbial engineering has been extensively studied for pharmaceutical applications, significant gaps remain in the comprehensive and systematic integration of advanced technologies such as AI, ML, and adaptive evolution into microbial biotechnology. Additionally, the ethical and regulatory challenges of engineered microbes require further scrutiny to ensure safe and sustainable implementation. Addressing these issues requires interdisciplinary approaches that integrate genetic engineering, synthetic biology, systems biology, and bioinformatics to enhance strain productivity, improve production efficiency, and ensure safety and efficacy [14,15,16]. Furthermore, fostering close collaboration between academia, industry, and regulatory bodies is essential for streamlining development pipelines and accelerating the integration of microbial engineering technologies into pharmaceutical manufacturing systems [17,18]. This review provides a comprehensive analysis of recent advancements in engineered microbial species for pharmaceutical applications, examines the key strategies underpinning these developments, and explores prospective directions for future research and innovation in the field. It aims to address existing scientific gaps through a comprehensive analysis of recent advancements in microbial engineering, with a particular focus on emerging technologies, key scientific and practical challenges, and future development trajectories. This study explores the role of AI-driven optimization strategies, adaptive evolution approaches, and synthetic biology innovations in enhancing the efficiency of microbial systems for pharmaceutical applications. Furthermore, it examines the regulatory and bioethical considerations necessary for the widespread clinical and industrial implementation of these technologies. By providing a scientifically grounded perspective, this work contributes to the advancement of microbial engineering as a promising avenue for integrating innovative solutions into pharmaceutical biotechnology.

## 2. Innovations, Strategies, and Future Directions in Microbial Engineering

### 2.1. Genetic Engineering

Genetic engineering, utilizing recombinant DNA technology and molecular biology methods, has revolutionized the field of microbial engineering by enabling precise modifications to microbial genomes [19,20]. This approach allows for the optimization of biosynthetic pathways, the enhancement of metabolite production, and the introduction of novel functionalities into microorganisms, thereby facilitating the deeper understanding and control of complex biological systems and ushering in a new biotechnology era [21,22].

**Figure 1:**
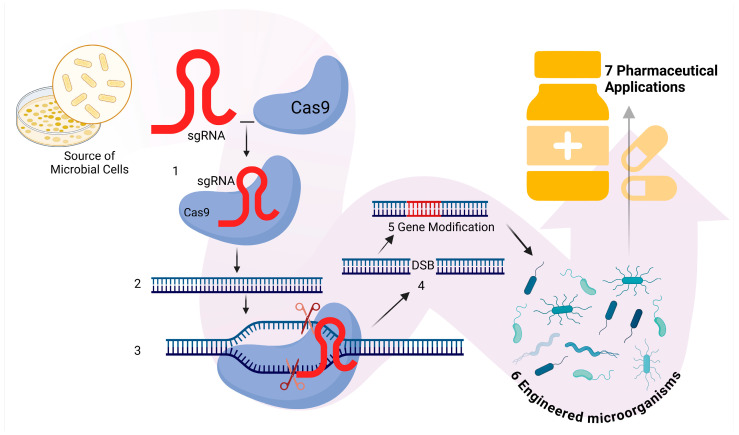
Application of CRISPR-Cas9 in engineering microorganisms for pharmaceuticals. (1) The sgRNA (single guide RNA) binds to the Cas9 enzyme. (2) The bound guide RNA and Cas9 enzyme search for the target DNA sequence within the cell. (3) The Cas9 enzyme unwinds the DNA and uses the guide RNA to identify the matching nucleotide sequence. (4) Cas9 introduces a double-strand break (DSB) at the target DNA site. (5) After the double-strand break, DNA repair mechanisms introduce the desired genetic modifications by incorporating an inserted sequence (in red), which is delivered via a donor DNA template through homologous recombination or non-homologous end joining. The efficiency of insertion can be affected by the size of the inserted sequence, with larger inserts generally being less efficient. (6) The genetically modified microorganisms are cultured, and their successful genetic alterations are verified. (7) These engineered microorganisms are utilized for pharmaceutical applications. Created with BioRender, License No. A12H414.

In genetic engineering, while Zinc Finger Nuclease (ZFN) technology has played an important role in gene editing, its application is limited by cytotoxic effects and high production costs [23,24]. Similarly, Transcription Activator-Like Effector Nuclease (TALEN) technology is distinguished by its high precision and minimal off-target effects, but its complex and resource-intensive modular assembly makes it challenging to use [25,26]. In contrast, the CRISPR-Cas system offers broader target selection, simplicity in assembly, and fewer off-target effects, making it a more effective alternative. This system enables precise gene editing and modification, establishing itself as the most efficient tool for genetic engineering in industrial and pharmaceutical applications [27,28]. CRISPR-Cas9 technology operates through a well-defined and precise genome editing mechanism, making it one of the most powerful tools in modern genetic engineering [29]. First, a specific single-guide RNA (sgRNA) is designed to target a particular DNA sequence within the microbial genome. The sgRNA binds to the Cas9 protein, forming a ribonucleoprotein complex. This complex then identifies and interacts with the complementary DNA target sequence. Once bound, the Cas9 protein introduces a double-strand break (DSB) at the target site [30,31]. This precision-driven editing mechanism has paved the way for significant advancements in pharmaceutical applications. CRISPR-Cas9 has been successfully applied to optimize metabolic pathways in microorganisms, enhance the production of therapeutic proteins such as insulin, and activate dormant biosynthetic clusters to discover novel bioactive compounds, including antibiotics and vaccines [32,33]. These innovations continue to advance biopharmaceutical development and shape its future (Figure 1).

CRISPR-Cas systems are widely utilized in modern genetic engineering due to their high precision and efficiency. This system enables the targeted modification of specific genes within microbial genomes, facilitating the introduction of precise mutations, the removal of inhibitory regulatory elements, or the integration of novel biosynthetic pathways. Such genetic modifications enhance production efficiency while reducing metabolic burden [34]. For example, the CRISPR-Cas9 system has been utilized to genetically modify *Escherichia coli,* optimizing its protein expression framework to achieve increased yields of recombinant insulin [35]. A comprehensive review by Dong et al. [36] focuses on the development of CRISPR/Cas9 systems for *Escherichia coli*, emphasizing their capability to enable high-precision and efficient genome editing for applications in metabolic engineering and pharmaceutical development. With the advancement of CRISPR/Cas9 genome editing technology, progress in genetic engineering has significantly expanded the scope of microbial systems in pharmaceutical development [37]. For instance, a study by Alberti and Corre [38] explored innovative approaches to utilizing CRISPR/Cas9 systems for editing *Streptomyces* genomes, demonstrating their potential in the discovery of novel antimicrobial natural products. The study highlighted the development of several CRISPR/Cas9-based tools, including pCRISPomyces plasmids, which enable the efficient and precise manipulation of biosynthetic gene clusters (BGCs). These advancements not only enhance the production of bioactive compounds but also lay the foundation for optimizing microbial systems for therapeutic applications.

A recent study by Andrea Ameruoso and colleagues [39] demonstrated the application of CRISPR interference (CRISPRi) and CRISPR activation (CRISPRa) systems to activate dormant biosynthetic gene clusters in *Streptomyces venezuelae*. The research highlighted the effective use of CRISPRi to relieve gene repression and CRISPRa to specifically activate transcription, facilitating the discovery of novel natural compounds by precisely modulating gene expression. Additionally, CRISPR technology can be used to inactivate or delete repressors of biosynthetic pathways, further enhancing gene cluster activation. A review by Azeez et al. [40] provides a comprehensive analysis of the potential of CRISPR-Cas systems in pharmaceutical applications, particularly their role in precision genome editing for therapeutic development. The study highlights advancements such as the development of Cas protein variants to enhance specificity and reduce off-target effects. These innovations enable the optimization of microbial systems for the production of biopharmaceuticals, including vaccines and therapeutic proteins.

Genetic engineering has fundamentally transformed microbial engineering, with CRISPR-Cas systems standing out as a pivotal tool due to their exceptional precision, efficiency, and scalability [41]. Genetic engineering is advancing pharmaceutical biotechnology through CRISPR-based genome editing, enhanced antibiotic biosynthesis, and the development of personalized medicine. The activation of biosynthetic gene clusters enables the discovery of novel natural compounds, while recombinant protein production enhances the synthetic efficiency of insulin and therapeutic enzymes. The integration of AI and CRISPR technologies optimizes genetic modifications, facilitating targeted therapies and innovative strategies in precision medicine (Table 1) [42]. These technologies have facilitated the identification of novel bioactive compounds, the enhancement of biosynthetic pathways, and a significant increase in the production of therapeutic products, driving innovation in pharmaceutical development and expanding the potential of biopharmaceutical applications.

Despite its high specificity, the CRISPR-Cas9 system can induce off-target mutations due to sequence mismatches, chromatin accessibility, and DNA repair mechanisms [54]. To mitigate this issue, several innovative strategies have been developed, including optimized guide RNA (gRNA) design, high-fidelity Cas9 variants, and genome-wide off-target screening methodologies [55]. Recent studies have identified several challenges associated with the application of CRISPR-Cas9 in microbial cells. Yang et al. [56] highlighted key constraints of CRISPR-Cas9 in bacteria, including off-target mutagenesis and inefficient delivery mechanisms. The authors emphasized that employing high-fidelity Cas9 variants is crucial to improving gRNA specificity and minimizing unintended genomic modifications.

Several methodologies have been employed to reduce off-target effects. For instance, CIRCLE-seq (circularization for the in vitro reporting of cleavage effects by sequencing) is a high-precision screening technique that enables the genome-wide identification of potential off-target sites in an unbiased manner [57]. This approach enhances gRNA specificity and significantly reduces the likelihood of unintended mutations. Additionally, the pORTMAGE system, designed to minimize off-target mutagenesis, has been successfully applied for precise genetic modifications in Escherichia coli [58]. This method utilizes a dominant-negative mutant protein to transiently suppress DNA repair pathways, thereby enabling more precise genomic alterations [59].

The integration of these advanced methodologies contributes to mitigating off-target mutagenesis in CRISPR-Cas9 technology and facilitates the efficient engineering of microbial strains. These strategies enhance the precision and reliability of genetic engineering applications in biomedicine, pharmaceutical production, and biotechnology, ultimately unlocking new therapeutic and industrial opportunities [60,61].

### 2.2. Synthetic Biology

Synthetic biology is an interdisciplinary field that integrates the principles of engineering and biology to design and construct new biological parts, devices, and systems, or to reprogram existing ones [62]. It plays a pivotal role in microbial engineering by enhancing the efficiency, scalability, and functionality of microorganisms for pharmaceutical applications. The ability to redesign gene expression to create minimal-genome microorganisms with novel traits or custom genetic circuits offers promising solutions for pharmaceutical applications, bioremediation, and biofuel production [63,64].

The robust theoretical foundation of synthetic biology enables researchers to integrate protein engineering, DNA synthesis, and in silico design methods to construct metabolic pathways and biological circuits that control cellular processes. These technologies not only improve communication systems between microorganisms but also expand their functionality through intercellular signaling and cooperative microbial networks [65].

A key aspect of synthetic biology is the availability of modular toolboxes that provide standardized genetic elements to control and optimize gene expression. These toolboxes include various types of promoters (constitutive and inducible), ribosome binding sites (RBSs) for translational efficiency, riboswitches that enable the dynamic regulation of gene expression in response to specific metabolites, and terminators ensuring proper transcriptional control. Additionally, synthetic biology toolkits often incorporate modular operons, genetic logic gates, and regulatory circuits that allow the precise manipulation of microbial metabolism. These elements are crucial for fine-tuning biosynthetic pathways and optimizing microbial production systems in pharmaceutical applications [66].

Through the development of genetic platforms and control systems, synthetic biology has transformed microorganisms into tools for diagnosing diseases and precisely delivering therapeutic molecules. Engineered microbes have been successfully applied in biosensors and diagnostic platforms, enabling the rapid and sensitive detection of pathogens and disease markers [67]. Engineered microbes overcome the limitations of traditional therapies by delivering therapeutic molecules in targeted, localized doses, minimizing side effects while maintaining efficacy. For example, microorganisms like *Bacteroides thetaiotaomicron*, capable of surviving in the human gut for extended periods, can be reprogrammed for the long-term monitoring and treatment of chronic diseases. Thus, synthetic biology is emerging as a powerful approach for reengineering microorganisms for pharmaceutical applications [68].

Synthetic biology has introduced a transformative shift in drug production by engineering microbes for efficient biosynthesis. For example, Jay Keasling and his colleagues [69] successfully modified *Escherichia coli* and *Saccharomyces cerevisiae* microorganisms to produce artemisinic acid, a precursor to the antimalarial drug artemisinin. By optimizing biosynthetic pathways and balancing metabolic processes, the researchers developed economically viable alternatives to plant-derived artemisinin, clearly demonstrating the transformative potential of synthetic biology in pharmaceutical production [70].

Synthetic biology has significantly improved the efficiency of pharmaceutical production by optimizing microbial systems for the biosynthesis of bioactive compounds. Rojo and colleagues [71] highlighted the advantages of engineered *Escherichia coli* and *Saccharomyces cerevisiae* microorganisms over traditional plant-based systems, demonstrating their ability to achieve higher yields and save time in addressing challenges in the production of compounds such as pterocarpans and coumestans. This study emphasized the potential of microbial engineering to overcome limitations inherent to plant-based systems, such as low yields and lengthy production cycles.

By employing synthetic genetic circuits, bacteria can be adapted to detect and respond to specific pathogens. For example, genetically modified *E. coli* bacteria act with high precision through engineered bacteriocins that inhibit the growth of *Pseudomonas aeruginosa.* These methods not only address the issue of antibiotic resistance but also improve the efficiency and accuracy of microbial systems [72]. The advancements in synthetic biology have also provided significant momentum for engineering probiotics and other members of the human microbiome for biomedical applications [73]. Modified microorganisms are being developed as biodiagnostic and therapeutic tools to address health challenges such as treating gastrointestinal disorders and regulating the immune system. Moreover, researchers have adapted microorganisms to produce antimicrobial peptides to combat antibiotic-resistant pathogens. For instance, *Lactobacillus* species have been engineered to detect multidrug-resistant *Enterococcus faecium* and release antimicrobial peptides against it. This highlights the immense potential of targeted microbial therapies [74].

Furthermore, synthetic biology has significantly improved the efficiency of pharmaceutical production by enabling the precise regulation of metabolic pathways and the optimization of genetic circuits. For example, data-driven models integrated with CRISPR-Cas systems have been successfully applied to reprogram metabolic fluxes in yeast, significantly enhancing the production of bioactive compounds [75]. This achievement exemplifies the successful integration of computational methods and advanced genetic editing technologies for designing and optimizing synthetic pathways, demonstrating great potential for the scalable biomanufacturing of pharmaceuticals.

Synthetic biology has enabled the reprogramming of microorganisms for the production of therapeutic compounds and other applications. By integrating chemical sensors with metabolic pathways, engineered microbes can detect specific molecules and dynamically regulate production efficiency. For instance, sensor-effector systems allow the real-time control of bioactive compound synthesis, facilitating the scaling of pharmaceutical production while maintaining precision and productivity [76]. These innovations highlight the critical role of synthetic biology in advancing microbial engineering.

The costs associated with research and development in synthetic biology have significantly increased in recent years. For instance, global investments in synthetic biology reached USD 9 billion in 2021. This funding has been directed toward developing new therapeutic tools, advancing gene engineering methods, and optimizing production processes [77,78]. Such investment levels highlight the rapid growth of synthetic biology and its establishment as a strategic direction in biotechnology. One of the most prominent examples of synthetic biology’s efficiency in pharmaceutical production is the development of mRNA vaccines. Pfizer and Moderna utilized advanced synthetic biology tools to create vaccines against the COVID-19 pandemic. These vaccines were developed in record time by reprogramming genetic information at the DNA and RNA levels, proving to be significantly faster and more efficient than traditional vaccine production methods [79]. This innovation underscores the transformative role of synthetic biology in modern biotechnology. Synthetic biology also has a substantial impact on the bioeconomy. In 2022, the global biomanufacturing market was valued at USD 1 trillion, with projections suggesting that it could exceed USD 2 trillion by 2030 [80]. This technology is widely applied across various sectors, from pharmaceutical products to biofuels, bioplastics, and food production, clearly demonstrating its vast economic potential.

Synthetic biology offers innovative approaches to reprogramming microorganisms for the production of pharmaceutical compounds. Its advancements have overcome the limitations of traditional production methods, significantly enhancing the efficiency of bioactive compound synthesis.

### 2.3. Adaptive Evolution

Adaptive evolution is an effective and complementary strategy for equipping microorganisms with desired traits by promoting their adaptation to specific environmental conditions over extended periods. Unlike direct genetic modifications, adaptive evolution leverages natural evolutionary processes, allowing the accumulation of spontaneous genetic changes that result in beneficial phenotypes [9,81,82].

In recent years, the application of adaptive evolution in microbial engineering has advanced significantly. The integration of high-throughput sequencing and omics technologies has enabled the monitoring and detailed characterization of genetic and phenotypic changes arising during evolutionary experiments. This approach has proven particularly effective in improving resistance to challenging conditions often encountered in pharmaceutical production, such as high temperatures, toxic byproducts, or extreme pH levels [9,83,84].

Recent studies have highlighted the effectiveness of adaptive evolution in enhancing microbial resistance to industrial stressors, particularly high temperatures. For example, Bailey and colleagues [85] conducted adaptive laboratory evolution (ALE) on *Escherichia coli* cultures by exposing them to elevated temperature conditions for 150 days. As a result, strains with significantly improved thermotolerance were obtained, with a maximum growth temperature approximately 2 °C higher than that of the parental strain. Moreover, the evolved strains retained stable recombinant protein production, demonstrating their high potential for pharmaceutical applications. In this context, ALE has been successfully utilized to enhance acid stress tolerance in *E. coli* and increase the production of valuable metabolites such as succinic acid. This method has been recognized as a critical tool for developing highly efficient and robust microbial strains for industrial applications. However, its time-intensive nature and the challenges associated with scaling up for industrial use remain key areas for future research and optimization [86]. Similarly, Yao and colleagues [87] successfully implemented the development of a furfural-tolerant *Saccharomyces cerevisiae* strain by combining ALE and CRISPR/Cas9 technology. Furfural, a common inhibitor in lignocellulosic hydrolysates, significantly impairs microbial growth and ethanol production. The study revealed that the ADR1_1802 mutant strain reduced lag phase duration by 20 h compared to the reference strain (*S. cerevisiae* CEN.PK113-5D) in the presence of 4 g/L furfural. The increased transcription levels of GRE2 (53.69%) and ADH6 (44.95%) indicated that accelerated furfural degradation was the primary mechanism of tolerance. This research highlights the significant potential of integrating adaptive evolution and genome editing techniques to enhance microbial performance for bioethanol production and other industrial applications.

Jahn and colleagues [88] demonstrated the high efficacy of ALE in studying antibiotic resistance in *Escherichia coli*. By exposing populations to prolonged selection with antibiotics such as amikacin, piperacillin, and tetracycline, the study revealed that the evolved strains achieved significantly enhanced resistance, with amikacin-tolerant strains surviving concentrations 170 times higher than the wild type. Despite variations in selection methods, consistent mutations in key genes such as *acrR* and *fusA* were observed. This highlights ALE’s potential in uncovering the genetic mechanisms of antibiotic resistance and its applicability in both clinical and industrial settings. Furthermore, Liu and colleagues [89] significantly improved the industrial performance of the probiotic *Bacillus coagulans* by combining ARTP mutagenesis with ALE. The resulting artp-aleBC15 mutant exhibited enhanced tolerance to acidic conditions (pH 2.5) and bile salts (0.3%), while maintaining stable cell morphology and improved membrane properties. These advancements position the mutant strain as a highly promising candidate for industrial applications. Adaptive evolution enhances microbial resilience and performance. Recent advances in sequencing and genome editing have improved their efficiency for developing robust microbial platforms.

### 2.4. Artificial Intelligence and Microbial Engineering

Artificial intelligence and machine learning play a crucial role as transformative tools in pharmaceutical research and microbial engineering. These technologies enable the analysis of large datasets, the optimization of metabolic pathways, and the development of predictive models [90]. AI enhances pharmaceutical innovations by improving processes such as drug development, virtual screening, and modeling interactions between drugs and target molecules. For example, ML and deep learning (DL) methods increase the efficiency of developing new drug molecules, such as biologics, while reducing costs [91]. Additionally, AI has significant potential in predicting toxicological risks and assessing safety levels, ensuring the stability and effectiveness of pharmaceutical compounds.

**Figure 2:**
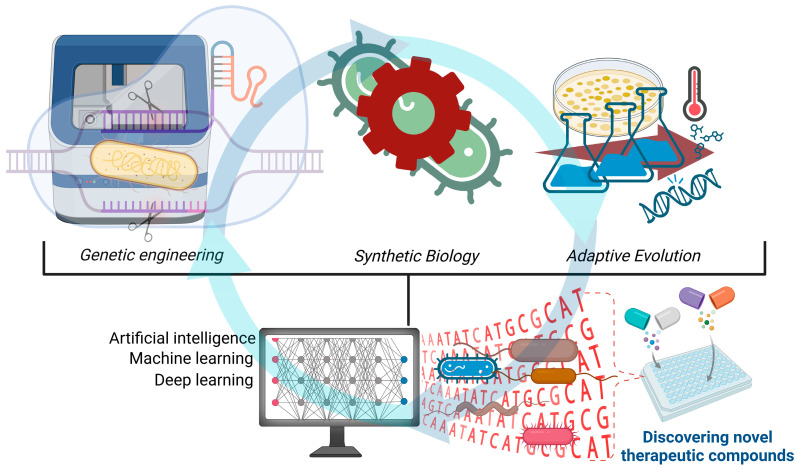
Integration of advanced technologies in microbial engineering for pharmaceutical applications. Genetic engineering, synthetic biology, and adaptive evolution, combined with AI and ML methods, enable the optimization of microbial strains, the comprehensive analysis of genetic data, and the advancement of drug discovery technologies. These approaches open new opportunities for the identification and production of highly effective therapeutic compounds, such as antibiotics, biopharmaceuticals, and vaccines. Created with BioRender, agreement No. PK27ZEC4QO.

In microbial engineering, AI and ML significantly enhance the processing of omics data, the identification of key genetic targets, and the design of optimized microbial strains. These technologies pave the way for the development of highly efficient and sustainable microbial systems for pharmaceutical production [92,93] (Figure 2).

Stokes et al. [94] demonstrated the transformative potential of AI and ML in the discovery of antimicrobial compounds. Using DL methods, they identified the antibiotic Halicin from a dataset of over 2000 compounds, which showed effectiveness against multidrug-resistant pathogens such as *Acinetobacter baumannii* and *Clostridioides difficile*. Applying this model to large chemical libraries like ZINC15 enabled the identification of structurally unique, broad-spectrum antimicrobial compounds, highlighting DL’s ability to accelerate therapeutic discoveries and explore new chemical spaces. Jiang et al. [95] emphasized the importance of advanced ML methods, such as Random Forest and neural networks, in microbiome classification and the discovery of microbial interactions [96].

AI and ML tools play a crucial role in pharmaceutical applications. For example, advanced tools like VirFinder enhance the accuracy of pathogen identification in metagenomic datasets, further strengthening AI and ML’s role in pharmaceutical research [97,98]. Recent advancements have significantly improved the accuracy of predicting CRISPRi guide efficiency. Yu et al. [99] developed an ML model to optimize CRISPRi targeting efficiency in bacterial systems. By integrating gene- and guide-specific features, this model achieved high precision and provided interpretable rules for selecting target sites near transcription start regions. Additionally, breakthroughs in DL, particularly transfer learning, have significantly impacted microbial classification. Wu and Gadsden [100] demonstrated that the DenseNet-121 model could classify 33 bacterial species in the DIBaS dataset with 99.08% accuracy. Their study highlighted the benefits of using pre-trained models based on large datasets like ImageNet and applying data augmentation techniques.

AI and ML have revolutionized forensic microbiology, enabling applications in personal identification, age and gender prediction, and environmental monitoring [101]. Advanced models such as Random Forest and deep convolutional neural networks have achieved high accuracy in analyzing microbial markers, while the SourceTracker tool has improved efficiency in identifying environmental microbiomes [102]. These technological advancements have significantly enhanced the accuracy and scope of microbial forensics, expanding its impact across various fields. Additionally, DL methods such as generative adversarial networks (GANs) and variational autoencoders (VAEs) have become essential tools for generating synthetic microbial genomic data and studying resistance mechanisms. These approaches not only improve predictive accuracy but also accelerate the discovery of new antibiotics and antimicrobial peptides, driving innovation in microbial engineering and drug development [103]. Alowais et al. [104] highlighted the transformative impact of AI and ML technologies in drug development and precision medicine. They emphasized the importance of AI-driven predictive models for optimizing drug dosages and monitoring adverse effects. For example, the CURATE.AI platform demonstrated the ability to dynamically adjust chemotherapy dosages for late-stage cancer patients, significantly improving treatment outcomes. Moreover, ML algorithms play a crucial role in predicting drug efficacy and toxicity, reducing clinical trial costs, and increasing success rates. These advancements underscore the vital role of AI and ML in developing personalized therapies and addressing complex medical challenges [105].

ML algorithms enhance metabolic pathway prediction and microbial modeling, improving the precision of genetic engineering. AI-CRISPR integration boosts gene editing efficiency and facilitates targeted strain modifications [106,107,108]. Deep learning accelerates the discovery of antibiotics, vaccines, and bioactive compounds. AI tools such as AntiSMASH and NPClassifier refine biosynthetic gene cluster identification and metabolite classification [109]. The integration of Raman spectroscopy enhances diagnostic accuracy, while AI-powered image recognition plays a crucial role in identifying antibiotic-resistant strains [110,111,112]. Millions of potential antibiotic compounds have been identified, with some demonstrating high efficacy against pathogens [113].

Woo et al. [114] developed explainable machine learning models (combining an elastic net regression and a deep neural network) to analyze *E. coli* metabolic networks. Their model learned patterns from gene knockout and metabolic flux data to pinpoint which metabolic reactions significantly influence bacterial growth on different carbon sources. Zhang et al. [115] integrated a genome-scale metabolic model with machine learning to optimize the tryptophan biosynthesis pathway in yeast. Their hybrid approach enabled accurate genotype-to-phenotype predictions and guided strain designs that achieved ~74% higher tryptophan titers and 43% higher productivity than the best prior designs.

Recent advancements in microbial engineering have unlocked significant opportunities in pharmaceutical, industrial, and environmental applications. Innovations in genetic engineering, synthetic biology, adaptive evolution, ML, and high-throughput screening have led to substantial progress in optimizing microorganisms for the efficient production of complex biological and chemical compounds. Despite these achievements, challenges and limitations remain in adopting and advancing these technologies, which are discussed in detail in the Challenges and Limitations section of this review.

### 2.5. Systems Biology in Microbial Engineering

The construction of beneficial microbial species for the pharmaceutical industry is one of the key directions in biotechnology [116]. For this process to be successful, a comprehensive analysis and management of the genetic and metabolic systems of microorganisms are required, which can be achieved through the tools of systems biology. Systems biology helps to model, optimize, and engineer microorganisms for pharmaceutical applications [117,118]. It analyzes how microorganisms function at the genomic, transcriptomic, proteomic, and metabolomic levels and suggests the most effective strategies to enhance their productivity [119].

Systems biology enables a comprehensive analysis of the complex interactions within microbial cells by mapping and modeling their metabolic networks. Through this approach, researchers can gain a deeper understanding of biosynthetic pathways and identify engineering strategies and potential limiting factors to optimize the production of pharmaceutically important compounds [120]. For example, the *Rhodobacter sphaeroides* strain has been genetically engineered to enhance the production of medically and industrially valuable compounds. Its metabolic networks were optimized using genome-scale metabolic modeling and CRISPR/Cas9 gene editing technology. As a result, the synthesis of target metabolites was significantly improved, achieving higher yields. In metabolic engineering, flux balance analysis (FBA) allows researchers to quantitatively analyze and optimize metabolic networks [121,122]. This approach helps identify effective genetic modification strategies to enhance the production of antibiotics, amino acids, and other biologically important compounds [123].

The integration of systems and synthetic biology enables the precise design of new genetic circuits and regulatory elements. This combination optimizes the biosynthesis of complex pharmaceutical compounds and facilitates the efficient construction of microbial strains with targeted properties [124]. Sheng et al. [125] developed a data-driven predictive model for CRISPR-based transcriptional regulation, allowing the programmable control of metabolic fluxes in yeast. This approach enables the precise modulation of gene expression, contributing to the optimization of biosynthesis for pharmaceutically important compounds.

Systems biology plays a crucial role in the development of live bacterial therapeutics, as it allows researchers to optimize drug delivery systems and precisely engineer microbial strains with enhanced therapeutic properties through in-depth analysis and the modeling of microbial behavior [126]. This approach has led to the creation of a new class of live biotherapeutics, consisting of engineered microbes designed to regulate specific disease mechanisms. By leveraging systems biology methods to study disease pathways, these engineered strains can modulate pathological processes and provide effective treatment strategies. Additionally, advancements in synthetic biology, chemistry, and nanotechnology have contributed to the development of bacterium-based drug delivery systems with improved tumor-targeting capabilities [127,128]. By enhancing their ability to sense and respond to the tumor microenvironment, these systems enable the precise and efficient delivery of anti-cancer drugs, thereby enhancing therapeutic efficacy [129].

To gain a deeper understanding of microbiome properties, systems biology widely employs meta-omics, computational modeling, and data integration methods [130]. These approaches facilitate the study of complex microbiome interactions and enable precise engineering to enhance or introduce novel functional traits for pharmaceutical applications [131]. Leggieri et al. [132] investigated how the integration of systems and synthetic biology contributes to analyzing the spatial and temporal dynamics of microbiomes. They emphasized the importance of this approach in designing microbiomes with enhanced or novel therapeutic properties. Their study highlighted the necessity of meta-omic analysis and computational modeling to gain deeper insights into microbiome interactions at the cellular and systemic levels [133]. Similarly, Dahal et al. [134] demonstrated the significance of integrating meta-omics and computational modeling in systems biology for studying microbiome interactions. Their research utilized genome-scale models to incorporate metagenomics, metatranscriptomics, and metabolomics data, showing how these approaches refine and reconstruct microbial community models. This method allows for a more detailed understanding of microbiome dynamics and helps characterize their functional potential [135,136].

## 3. Challenges and Limitations

### 3.1. Antimicrobial Resistance

The emergence and global spread of antimicrobial resistance (AMR) represent one of the most urgent public health challenges today. This issue is particularly relevant to the field of engineered microbial therapeutics, where the use of antibiotic resistance markers during strain development and the potential for HGT raise significant concerns. If resistance determinants inadvertently disseminate from engineered microbes to pathogenic bacteria, the efficacy of current antibiotics could be undermined, exacerbating the AMR crisis [137].

Antibiotic resistance genes have traditionally been used as selection markers during the genetic modification of microorganisms. While these markers greatly facilitate the engineering process, they also pose a risk of transferring resistance traits to non-target organisms. The probability of HGT increases when engineered microbes are introduced into the human body, where they coexist with complex native microbial communities. Such gene transfer could occur via conjugation, transformation, or transduction, potentially seeding resistance in otherwise susceptible pathogens [137].

To address these risks, researchers are actively developing marker-free genetic engineering strategies. Advances in CRISPR-Cas genome editing have enabled the creation of modified strains without the permanent incorporation of antibiotic resistance genes. In addition, metabolic selection methods—where the growth of engineered strains is tied to the expression of a desirable phenotype—offer alternative routes for strain development that avoid reliance on antibiotic markers [138]. These approaches are increasingly important as the field seeks to minimize the risk of inadvertently contributing to the AMR problem.

Beyond selection strategies, biocontainment systems play a vital role in mitigating AMR risks. Engineered kill-switches and auxotrophy-based systems are designed to limit the survival and proliferation of modified organisms outside of controlled environments. By ensuring that engineered microbes cannot persist in the natural environment or in the host after they have served their therapeutic purpose, these systems help to contain any resistance genes that may be present [132,138]. Although promising, these biocontainment strategies must be validated under industrial conditions to guarantee their robustness and long-term effectiveness.

The regulatory landscape is also evolving to address the potential contribution of engineered microbial therapeutics to the AMR crisis. Agencies such as the FDA and EMA now require detailed assessments of the risk of horizontal gene transfer and the presence of antibiotic resistance markers in regulatory submissions for live biotherapeutic products [139]. Such assessments are critical for ensuring that the benefits of microbial therapeutics do not come at the expense of increasing antimicrobial resistance in the broader environment.

The public health implications of AMR extend well beyond the laboratory. Reports from organizations like the Centers for Disease Control and Prevention (CDC) [138] and the World Health Organization (WHO) [139] underscore the urgent need for innovative strategies to combat resistant infections. In this context, the development of engineered microbial therapeutics must proceed with caution, balancing the potential benefits of these novel treatments against the risk that they could inadvertently contribute to the global AMR crisis [140].

In addition to microbial contaminants, genetic contamination poses a significant risk. Engineered microbes are constructed with specific genetic modifications to confer desired therapeutic properties. However, these genetic elements—often including selection markers such as antibiotic resistance genes—may inadvertently be transferred to other microorganisms through HGT [140]. Such events could lead to the spread of unwanted traits in environmental or host-associated microbial communities, potentially compromising not only the therapeutic product but also contributing to broader biosafety concerns [133].

To mitigate genetic contamination, researchers have developed innovative biocontainment strategies. For instance, engineered kill-switches can be incorporated into microbial genomes so that the organism self-destructs when exposed to conditions outside the controlled production environment [140,141]. Although these strategies show promise in laboratory settings, demonstrating their reliability in large-scale production remains a critical hurdle. The robust validation of these systems is necessary to ensure that they function consistently and do not inadvertently trigger premature cell death, which could affect product yield and efficacy [142].

Environmental release is another dimension of contamination risk. Accidental dissemination of engineered microbes could have unforeseen ecological impacts. To prevent this, stringent physical containment measures—such as closed-system bioreactors—and rigorous waste decontamination protocols are essential components of the manufacturing process [143]. Regulatory bodies require comprehensive environmental risk assessments as part of the approval process for live biotherapeutic products, underscoring the need for reliable contamination control strategies [67]. Table 2 provides concrete examples of microbial contamination events in biopharmaceutical manufacturing by listing specific organisms.

### 3.2. Contamination Risks

**Figure 3:**
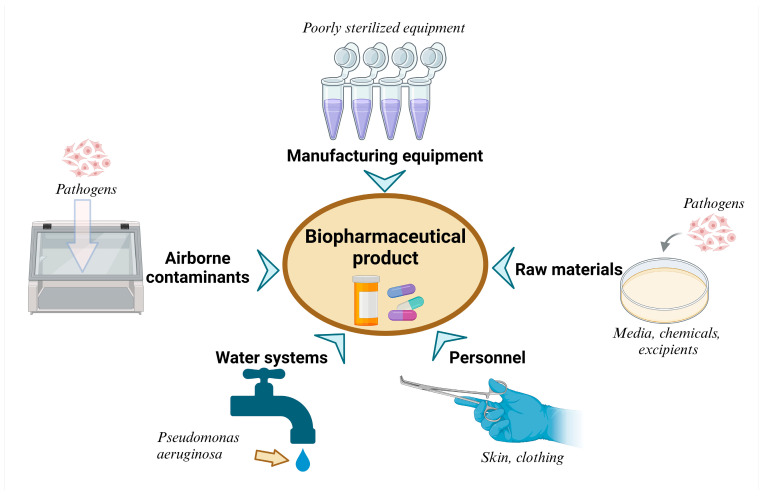
Sources of microbial contamination in biopharmaceutical manufacturing [144,145]. Created with BioRender, agreement No. KV27WPI3T0.

The production and use of engineered microbial species for pharmaceutical applications are inherently susceptible to various forms of contamination. Contamination risks can manifest during the genetic engineering phase, in laboratory-scale culture, throughout large-scale bioprocessing, and even during the final product formulation. Both microbial and genetic contaminations present serious challenges that can compromise product quality, efficacy, and patient safety. Figure 3 shows the various sources of microbial contamination in the production of engineered microbial products, highlighting potential pathways of contamination and mitigation strategies to ensure product safety and regulatory compliance.

Microbial contamination is one of the most persistent challenges in biopharmaceutical manufacturing. Even under strictly controlled aseptic conditions and rigorous adherence to good manufacturing practices (GMPs), the introduction of adventitious agents—bacteria, fungi, or viruses—can occur through raw materials, process water, or human contact [146]. In large-scale fermentations, nutrient-rich environments can facilitate the rapid growth of contaminants, leading not only to product loss but also to safety risks if these contaminants survive downstream purification processes. The high cost and complexity of implementing comprehensive environmental monitoring systems further exacerbate these risks, especially for smaller enterprises or academic laboratories.

To counteract these challenges, advanced analytical methods have been developed to detect contamination early in the production process. Techniques such as quantitative polymerase chain reaction (qPCR), next-generation sequencing (NGS), and mass spectrometry are increasingly integrated into manufacturing workflows, allowing for real-time monitoring and rapid intervention when contamination is detected [135]. Despite these advances, the need for such sophisticated systems increases both the capital and operational costs associated with manufacturing engineered microbial therapeutics.

### 3.3. Regulatory Barriers

The development of engineered microbial species for pharmaceutical applications faces substantial regulatory challenges (Table 3). Regulatory agencies such as the U.S. Food and Drug Administration (FDA) and the European Medicines Agency (EMA) have issued guidance documents addressing live biotherapeutic products [147,148]. However, the rapid evolution of synthetic biology and microbial engineering has outpaced many traditional regulatory frameworks. Engineered microbes—designed to deliver drugs, modulate metabolic pathways, or even sense and respond to disease signals—do not fit neatly into the existing categories established for small-molecule drugs or conventional biologics [149,150].

One central challenge is the lack of harmonized definitions and risk assessments across different jurisdictions. In the United States, the FDA employs risk-based approaches that evaluate safety, efficacy, and manufacturing consistency. Yet, the inherent complexity of live microbial systems—especially those capable of self-replication—raises concerns about long-term safety and environmental impact [129]. In contrast, the EMA has traditionally taken a more precautionary stance, requiring extensive non-clinical data and environmental risk assessments before granting market approval [130]. This divergence means that a microbial therapeutic that meets U.S. regulatory requirements may still face significant hurdles in Europe, leading to prolonged preclinical studies and additional clinical trial phases.

Another layer of complexity arises from the classification of these products. Engineered microbes may be considered drugs, biologics, or even gene therapy products, depending on their mode of action and manufacturing processes. For example, products that employ genetically modified organisms (GMOs) to secrete therapeutic proteins have been variously categorized, sometimes resulting in overlapping review processes and uncertainty over the applicable regulatory pathway [151]. Such ambiguity not only delays the approval process but also increases the overall cost of product development.

Safety is at the forefront of regulatory concerns. Engineered microbes are designed to interact with complex host systems, and their in vivo behavior can be difficult to predict. Concerns include the possibility of horizontal gene transfer (HGT) to native microbiota, the emergence of unintended immunogenic responses, and the potential for long-term colonization that might alter the host’s microbial balance [131]. Even with rigorous preclinical models, these risks necessitate extensive and often costly post-market surveillance programs to monitor adverse outcomes after product approval.

Manufacturing quality control further complicates the regulatory picture. The production of live microbial therapeutics must adhere to strict GMPs, and ensuring consistency from batch to batch is challenging given the dynamic nature of biological systems. Advances in metabolic engineering and synthetic biology have enabled remarkable product innovations, yet even slight variations in culture conditions or genetic stability can lead to significant differences in product performance [152,153]. Regulators now demand the detailed validation of manufacturing processes, a requirement that has pushed companies to invest heavily in advanced monitoring and quality assurance systems.

**Table 3:** Regulatory barriers associated with engineered microbial therapeutics.

Regulatory Barrier	Description	Impact on Development	Mitigation Strategies	Reference
Product classification	Ambiguity in categorizing engineered microbes	Divergent review processes and approval delays	Harmonized definitions	[115,116,151]
Risk assessment	Extensive preclinical and post-market monitoring required due to unpredictable in vivo behavior and potential off-target effects	Increased development costs and extended timelines	Risk-based approaches; improved in vivo models and rigorous post-market surveillance	[115,116,117]
Manufacturing	Challenges ensuring batch-to-batch consistency in live microbial production given inherent biological variability	Variability in product quality and potential for process-related rejections	Advanced GMPs and real-time monitoring systems	[152]
Environmental impact	The potential for horizontal gene transfer and unintended environmental release raises significant biosafety concerns	Can lead to increased regulatory scrutiny and the need for extensive environmental risk assessments, potentially delaying product approval	Develop robust biocontainment strategies	[149]
International variability	Regulatory requirements vary widely across regions, complicating product development	Complicates the design of global clinical trials and market access strategies, leading to additional time and cost burdens	Promote international harmonization and collaboration among regulatory agencies	[115,116,154]

Moreover, the ethical and public policy dimensions of releasing genetically modified organisms (GMOs) into clinical and environmental settings have added an extra layer of scrutiny. The public perception of GMOs influences regulatory policies, and differing cultural attitudes toward genetic modification can lead to disparate national requirements [154]. In response, some industry groups and regulatory bodies have initiated collaborative efforts—through workshops and public–private partnerships—to develop adaptive regulatory frameworks that balance innovation with safety.

**Figure 4:**
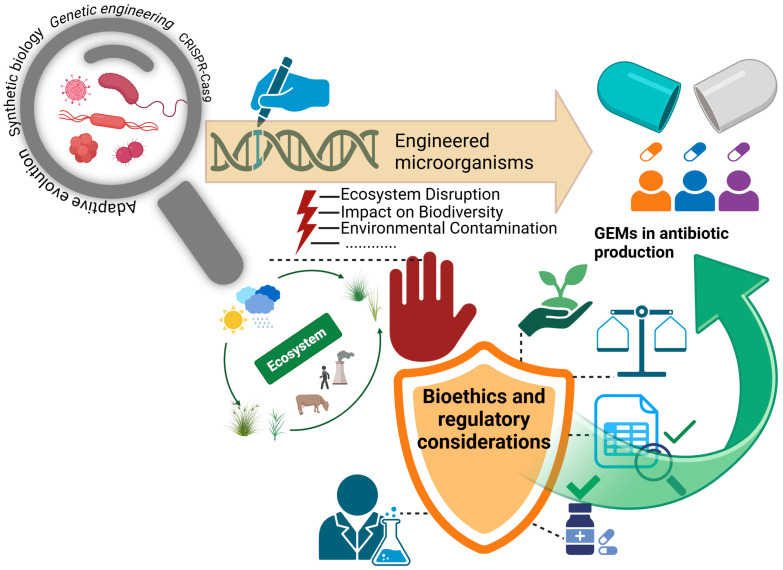
Bioethics and regulatory considerations in the application of GEMs. The figure illustrates the role and potential of GEMs in antibiotic production. It also highlights their potential ecological risks, including ecosystem imbalance, impact on biodiversity, and environmental contamination. The importance of bioethical principles and regulatory mechanisms in ensuring the safe and sustainable application of these technologies is emphasized. Created with BioRender, agreement No. TL27WPHN14.

Genetically engineered microorganisms (GEMs) are increasingly being utilized in the pharmaceutical industry, offering significant advantages in the production of medicinal and therapeutic compounds [155]. However, the use of GEMs in the development of microbial species for pharmaceutical applications raises a number of complex ethical and regulatory issues [156,157]. To ensure the safe application of these technologies, comprehensively assess their potential environmental impacts, and enhance public acceptance, it is crucial to thoroughly analyze bioethical principles and legal frameworks while developing clear and effective solutions (Figure 4) [158,159].

GEMs have the potential to transfer genetic material to native organisms, facilitating the spread of phenotypic traits such as antibiotic resistance or toxin production [160]. These processes can disrupt the natural balance of ecosystems, leading to the displacement of native species, alterations in nutrient cycles, and, in some cases, the emergence of resistant forms of pests and pathogens [161,162].

Measures to mitigate risks include the use of plasmids incapable of transferring genetic information, biological containment strategies, and mechanisms to monitor the long-term viability of GEMs [163,164]. When developing GEMs for pharmaceutical applications, ensuring their safety and efficacy must be a primary focus. In the development of GEMs for pharmaceutical purposes, it is crucial to maintain genetic stability, restrict the horizontal transfer of genetic elements, and incorporate biological containment systems that allow for environmental monitoring and control [165,166]. GEM strains used in pharmaceutical production should be specifically optimized for the synthesis of target metabolites or bioactive compounds, thereby enhancing their safety profile and reducing environmental impact. Furthermore, it is essential to investigate the long-term interactions of GEMs with ecosystems, including their potential effects on biodiversity and pharmaceutical production systems. Future research should aim to improve pharmaceutical GEM strains through advanced tools like synthetic biology, CRISPR/Cas systems, and systems biology, ensuring that these microorganisms meet both safety and efficacy standards in pharmaceutical applications [167,168,169].

Efforts to streamline the regulatory process are ongoing. Recent initiatives aim to harmonize standards internationally and reduce redundant testing requirements (Table 3). For example, discussions between regulatory agencies and stakeholders in the synthetic biology community have led to proposals for unified risk assessment protocols that specifically address the unique characteristics of live microbial therapeutics [170,171]. Such collaborations are vital for ensuring that innovative therapies can reach patients without compromising safety [116,117].

#### International Regulatory Frameworks and Harmonization for GEMs

While the environmental risks and biosafety concerns of GEMs are global issues, regulatory approaches differ markedly across regions. The United States, European Union, and China have each developed distinct frameworks for the biosafety and environmental risk assessment of GEMs. This section compares these regional policies, highlights challenges in aligning them, and discusses efforts and strategies toward international harmonization.

The U.S. regulates GEMs under its Coordinated Framework for Biotechnology (est. 1986), which distributes oversight among the USDA, EPA, and FDA. This framework is product-focused and risk-based: GEMs are evaluated according to their intended use and potential risks rather than the method of creation. For example, the FDA applies the principle of “substantial equivalence”, treating foods or feeds from GEMs like conventional products unless they differ significantly in composition or safety. Environmental assessments are typically conducted by USDA (if the GEM could affect plants) or EPA (if the GEM functions as a pesticide or falls under toxic substance regulations). Overall, the U.S. approach emphasizes scientific risk assessment of the final organism’s traits, with fewer procedural hurdles if a GEM is deemed similar in risk to non-GE counterparts [137,172].

The EU follows a process-based and precautionary approach in regulating GEMs, treating any organism made with modern biotechnology as a GMO requiring case-by-case scrutiny. EU directives and regulations impose strict biosafety measures. For instance, Directive 2001/18/EC mandates a thorough environmental risk assessment before any deliberate release of a GMO (including GEMs) into the environment. Likewise, Regulation (EC) 1829/2003 requires comprehensive safety evaluations by the European Food Safety Authority (EFSA) for GEM-derived products used in food or feed, along with mandatory labeling for consumer transparency. The EU’s GMO framework also includes traceability requirements (Regulation 1830/2003) to monitor GEMs through the supply chain. Driven by the precautionary principle and public wariness, the EU’s stringent policies have resulted in relatively few approvals and strict controls on commercialization [138,173].

China maintains a highly controlled and biosafety-centric regulatory regime for GEMs, increasingly resembling the EU’s precautionary stance. The cornerstone is the Regulations on Safety of Agricultural GMOs (2001, amended 2017), which provide a comprehensive framework governing GMO/GEM research, development, testing, production, marketing, and import/export. The Ministry of Agriculture and Rural Affairs (MARA) oversees approvals through a rigorous biosafety evaluation process, and a national biosafety committee review risks before any GEM is approved. China also mandates the labeling of GMO products, including those involving GEMs, to inform consumers. These measures reflect China’s cautious approach focused on preventing environmental and health risks, with controlled field trials and the slow introduction of GEM applications. Notably, China is a party to the Cartagena Protocol and has integrated its principles into domestic law, moving from a more permissive stance in the 1990s to a precautionary, process-oriented system in line with international biosafety norms [139,174].

## 4. Conclusions

Microbial engineering is increasingly becoming a pivotal tool in pharmaceutical biotechnology, serving as a strategic platform for the discovery and biosynthesis of novel therapeutic agents. Advances in genetic engineering, synthetic biology, and metabolic pathway optimization have expanded the potential of microbial systems, enabling the targeted production of biopharmaceutical products such as antibiotics, biologics, and recombinant proteins. Furthermore, the integration of CRISPR-Cas systems and artificial intelligence technologies has significantly enhanced the precision of microbial strain optimization, elevating the efficiency and productivity of biopharmaceutical manufacturing. However, the clinical and industrial applications of microbial engineering remain constrained by several challenges, including complex regulatory requirements, biosafety concerns, and the growing threat of AMR. Ensuring the safe and purposeful application of engineered microbes necessitates the development of stringent biocontainment strategies and the refinement of international and national regulatory frameworks. Additionally, interdisciplinary collaboration among the scientific community, the industrial sector, and regulatory authorities is crucial for the effective advancement and implementation of these technologies. Future research should focus on expanding the metabolic capabilities of microbial systems, developing sustainable and environmentally friendly production processes, and improving the genomic stability of engineered microorganisms.

## Figures and Tables

**Table 1:** Future directions in microbial engineering for pharmaceutical applications.

Future Direction	Objective and Application Areas	Reference
CRISPR-based drug development	Precise treatment of genetic diseases and gene therapy, targeting rare genetic disorders	[43]
Cell and gene therapies	Correction of mutant genes, cell regeneration for cancer and hereditary diseases	[44]
Enhanced antibiotic production	Combating antibiotic-resistant bacteria, development of new antibiotics	[45,46]
Personalized medicine	Tailoring treatments based on patient genetics, precision pharmacotherapy	[47]
Biopharmaceutical active compounds	Increasing production of biologically derived drugs, vaccines, insulin, hormones	[48,49]
Activation of biosynthetic gene clusters	Discovery of novel natural compounds, antibiotics, antiviral drugs	[50]
Recombinant protein production	Artificial synthesis of essential human proteins, insulin, therapeutic enzymes	[51,52]
AI and CRISPR integration	Automating genome editing to accelerate new drug development	[53]

**Table 2:** Microbial contamination events in biopharmaceutical manufacturing.

Microorganism	Contamination Source	Product	Strategies	Reference
*Ralstonia pickettii*	Contaminated water systems used in bioreactors and manufacturing equipment	Can lead to product spoilage and endotoxin contamination; may cause batch failures	Enhanced water system sterilization; use of ultrafiltration and validated decontamination protocols; routine environmental monitoring	[140]
*Pseudomonas aeruginosa*	Environmental sources (air, surfaces, equipment) within manufacturing facilities	Biofilm formation on equipment surfaces; production downtime; potential endotoxin release affecting product safety	Strict facility hygiene practices; routine disinfection; installation of high-efficiency particulate air (HEPA) filtration and regular environmental monitoring	[141]
*Burkholderia* *cepacia complex*	Contaminated raw materials or water used in production processes	Leads to production delays, product recalls, and poses risks of patient infection due to its intrinsic resistance mechanisms	Rigorous quality control for raw materials and water; validated cleaning procedures; regular microbial testing of production environments	[142]
*Bacillus cereus*	Airborne spores entering sterile manufacturing areas during production	Potential for pyrogenic reactions; product contamination may lead to recalls and safety concerns	Implementation of HEPA filtration; strict environmental monitoring; effective sanitization protocols and controlled air-handling systems	[143]

## Data Availability

Data are available on request.

## Abbreviations

ADH6	Alcohol dehydrogenase gene
ADR1	A transcription factor involved in stress response
AI-CRISPR	Artificial intelligence-assisted CRISPR technology
ALE	Adaptive laboratory evolution
ANTI-SMASH	Antibiotic and Secondary Metabolite Analysis Shell
ARTP	Atmospheric and Room Temperature Plasma
BGC	Biosynthetic gene cluster
CDC	Centers for Disease Control and Prevention
CRISPR	Clustered Regularly Interspaced Short Palindromic Repeats
Cas	CRISPR-associated protein
DL	Deep learning
DNA	Deoxyribonucleic Acid
DOAJ	Directory of Open Access Journals
DSB	Double-strand break
EMA	European Medicines Agency
FDA	U.S. Food and Drug Administration
GAN	Generative adversarial network
GEM	Genetically engineered microorganism
GMP	Good manufacturing practice
GRE2	A gene involved in stress resistance
HEPA	High-efficiency particulate air (filtration)
HGT	Horizontal gene transfer
LD	Linear Dichroism
MDPI	Multidisciplinary Digital Publishing Institute
ML	Machine learning
NGS	Next-generation sequencing
RNA	Ribonucleic Acid
TALEN	Transcription Activator-Like Effector Nuclease
TLA	Three-Letter Acronym
VAE	Variational autoencoder
WHO	World Health Organization
ZFN	Zinc Finger Nuclease
mRNA	Messenger RNA
